# Healthcare consequences of FEMA's anticipated response limitations in 2025: a gathering storm for health systems

**DOI:** 10.1016/j.lana.2025.101163

**Published:** 2025-06-21

**Authors:** Attila J. Hertelendy, Gregory R. Ciottone

**Affiliations:** a Department of Information Systems and Business Analytics, College of Business, Florida International University, Miami, FL, USA; b Disaster Medicine Fellowship, Beth Israel Deaconess Medical Center, Department of Emergency Medicine, Boston, MA, USA; c Harvard Medical School, Boston, MA, USA

The Federal Emergency Management Agency (FEMA), established in 1979 under President Jimmy Carter, serves as the United States' operational resource for assisting in the coordination of disaster response when local and state capacities are overwhelmed. Operating under the Department of Homeland Security since 2003, FEMA's mission is to assist individuals and communities before, during, and after disasters. It represents the cornerstone of a response system that is designed to flex local, state, and federal assets as they are needed in large-scale disasters. FEMA provides individual assistance through temporary housing support, home repair funds, and financial aid for essential needs such as medical and childcare expenses, thereby helping residents restore stability following a disaster.^1^ For communities, FEMA offers public assistance programs, providing grants to local governments and organizations to repair damaged infrastructure, including roads, hospitals, and emergency services facilities, as well as support community recovery initiatives. Additionally, FEMA coordinates hazard mitigation funding aimed at improving community resilience through infrastructure enhancement, land-use planning, and adoption of building codes designed to reduce disaster vulnerabilities.^1^

In healthcare preparedness and response, FEMA plays a pivotal role within the National Response Framework, which outlines scalable and adaptable strategies for large-scale emergencies. The agency also works closely with the National Disaster Medical System (NDMS), which deploys Disaster Medical Assistance Teams (DMATs) that are comprised of healthcare professionals who provide critical medical care during large scale emergencies. Additionally, FEMA collaborates with public health entities to enhance emergency preparedness, emphasizing the integration of risk awareness and emergency planning. Contemporary disaster events, such as the March 2025 multi-state tornado outbreak resulting in over 40 casualties across Arkansas, Mississippi, and Missouri, where federal assistance was initially denied, illustrate the systemic challenges that state, local, tribal, and territorial jurisdictions encounter when operating without federal disaster relief mechanisms.^2^ The Los Angeles County wildfires of early 2025 exemplify comparable challenges, resulting in extensive property destruction and residential displacement. Despite over 24,000 disaster assistance applications submitted to federal agencies, widespread initial denials created systemic administrative inefficiencies and elevated psychosocial stress within affected communities.^2^^,^^3^

There is now a consideration to restructure FEMA, potentially to include eliminating the agency and shifting responsibility for disaster response to states.^4^^,^^5^ There's also a draft bill seeking to reform FEMA, focusing on streamlining processes and improving disaster relief.^6^ The agency is concurrently facing cuts in training and funding, leading to concern of the impact this will have on disaster preparedness. This all comes at a precarious time, with hurricane season just beginning.^4^^,^^5^

As the 2025 Atlantic hurricane season and concurrent wildfire season begins, reports suggest FEMA's waning capacity to respond to disasters may pose significant risks to public health infrastructure, exacerbating vulnerabilities in emergency medical care, hospital preparedness, and equitable resource distribution.^7^ An internal review obtained by CBS News revealed FEMA is “not ready” for hurricane season amid staff reductions exceeding 2000 employees (33% of its workforce) and the elimination of critical resilience programs.^8^ These systemic challenges threaten to amplify morbidity and mortality during disasters, particularly for vulnerable communities reliant on federal support.

FEMA's workforce attrition may limit its ability to coordinate rapid medical interventions. The agency's internal May 2025 assessment noted that preparedness efforts were “derailed” by staffing shortages and contract disputes, leaving disaster-stricken regions without timely aid.^7^ Such lapses may directly compromise the provision of healthcare for patients with chronic conditions facing interrupted treatments, and trauma victims experiencing prolonged waits for emergency services. The Department of Homeland Security's Office of Inspector General previously warned of FEMA's logistical failures during COVID-19, including medical supply shipment breakdowns resulting in hospitals lacking ventilators and PPE.^9^ In 2025, similar interruptions could negatively impact regional health systems during concurrent disasters.^10^

The healthcare consequences of FEMA's limited ability to respond are neither hypothetical nor inevitable. FEMA's mission must be sustained to ensure effective healthcare care delivery in times of crisis. The agency's decline could strain public health systems already grappling with chronic underinvestment. While it is true that all disasters require a robust local response, history provides ample examples of events that overwhelm regional and state resources, requiring federal assistance. If FEMA's response capacities were to be degraded, it would leave a void in that capability. Policymakers must recognize disaster preparedness as a cornerstone of national health security before the next crisis strikes.

## Contributors

AJH, led the project, provided oversight, reviewed the literature, wrote the first draft, and coordinated edits. GC contributed content, and edits.

## Declaration of interests

We declare no competing interests.
